# Cross-cultural adaptation and validation of a Moroccan Arabic version of the Hooked on Nicotine Checklist

**DOI:** 10.3389/fpubh.2026.1815960

**Published:** 2026-05-22

**Authors:** Salma Ghofrane Moutawakkil, Abdelfettah El-Ammari, Samir El Gnaoui, Karima El Rhazi, Btissame Zarrouq

**Affiliations:** 1 Laboratory of Epidemiology and Research in Health Sciences, Faculty of Medicine, Pharmacy, and Dental Medicine, Sidi Mohamed Ben Abdellah University, Fez, Morocco; 2 Laboratory of Scientific Innovation in Sustainability, Environment, Education, and Health in the Era of Artificial Intelligence, Teachers Training College (Ecole Normale Superieure), Sidi Mohamed Ben Abdellah University, Fez, Morocco; 3 Addictology Center, Fez, Morocco

**Keywords:** hooked on nicotine checklist (HONC), Morocco, nicotine dependence, psychometrics, reliability, tobacco, validity

## Abstract

**Background:**

Early identification of nicotine dependence symptoms is essential for tobacco control and prevention strategies. The Hooked On Nicotine Checklist (HONC) is widely used to assess early symptoms of nicotine dependence. This study aimed to translate, culturally adapt, and evaluate the psychometric properties of a Moroccan Arabic version of the HONC.

**Methods:**

A cross sectional study was conducted among a sample of 234 Moroccan smokers. The HONC was translated and adapted following established guidelines. Descriptive statistics and item–total correlations were examined. Internal consistency was assessed using Cronbach’s alpha and McDonald’s omega. Dimensionality was evaluated using confirmatory factor analysis (CFA) with the WLSMV estimator. IRT analyses were conducted using a two-parameter logistic (2PL) model. Assumptions of unidimensionality, monotonicity, and local independence were systematically tested. Item discrimination and difficulty parameters, item characteristic curves, and test information functions were estimated.

**Results:**

Item–total correlations were moderate-to-high (range: 0.45–0.77). Internal consistency was excellent (Cronbach’s *α* = 0.905; ωt = 0.908). The one-factor CFA showed good model fit (*χ*^2^/df = 1.34; CFI = 0.998; TLI = 0.998; RMSEA = 0.038; SRMR = 0.059), with substantial standardized loadings (0.629–0.952). Monotonicity and local independence assumptions were fulfilled. Discrimination parameters were generally high (a = 1.40–4.69) and difficulties were mostly negative (b ≈ −0.64 to 0.00), suggesting that the scale is most informative at lower to moderate levels of nicotine dependence.

**Conclusion:**

The Moroccan Arabic HONC is a reliable and valid measure of early nicotine dependence. It can be confidently used for research, screening, and tobacco control interventions.

## Introduction

Tobacco use is one of the main avoidable risk factors of non-communicable diseases. In 2020, 22.3% of the global population used tobacco, with nearly 5 times more prevalent use in men compared with women (36.7 and 7.8%, respectively). Around 80% of the 1.3 billion tobacco users worldwide reside in low- and middle-income countries, enduring, thus, the heaviest burden of illness and death linked to tobacco use (1). In Morocco, according to the latest STEPS survey report, 13.4% (12.2–14.6) of Moroccans, aged 18 and older, were current tobacco users. The prevalence was much higher in men (26.9%) than in women (0.4%) (2). These figures underscore the continuing public health importance of tobacco dependence assessment in Morocco, especially considering its implications for cessation, relapse prevention, and the tailoring of intervention strategies.

Tobacco use exposes its users to a wide spectrum of substances, including nicotine, the main chemically active component of tobacco, characterized by its highly addictive potential and considered as the major dependence producing agent, responsible for failure of quitting attempts and maintained use (3, 4). “Loss of autonomy” is a common feature among definitions of substance dependence. Based on this concept, the Hooked On Nicotine Checklist (HONC) was developed (5). The HONC is a theory-derived widely used measurement tool, consisting of 10 items that assess lost autonomy. Diffranza’s Autonomy Theory (5, 6) advances that the onset of dependence occurs when individuals are “hooked” or lose their autonomy over tobacco use (when quitting becomes difficult), and integrates three mechanistic theories of independence; the Self-Medication Theory (7), which postulates that dependence is the result of the use of psychoactive substances to “self-medicate” or to cope with negative symptoms associated with a mental health condition or other issues (HONC item 5), the Incentive-Sensitization Theory (8), which suggests that repeated PASs use renders neural system hypersensitive (‘phenomenon of ‘sensitization’). This hypersensitization leads to generation of “incentive salience” toward drugs and their associated stimuli which become highly salient and “wanted,” causing thus craving, and the Negative Reinforcement Theory, which posits that individuals continue to use drugs to alleviate uncomfortable emotional states, including those associated with withdrawal (9).

The original version of the HONC (5) has a dichotomous (Yes/No) response format, but a multiple response choices format was afterwards proposed by O’Loughlin et al. (10). Each HONC item reflects a ND symptom. Affirmation of any item (i.e., positive HONC) implies loss of full autonomy over tobacco use (diminished autonomy), while a negative HONC (a score of zero) means enjoying full autonomy over tobacco use and higher scores signify more severe loss of autonomy levels, which indicates greater ND. The original HONC has high internal consistency (=0.94) and good test–retest reliability (*kappa* = 0.61 (95% CI, 0.35–0.87)). Concurrent validity was supported by the statistically significant positive associations between the total number of HONC endorsed symptoms and the maximum amount smoked (*r* = 0.65, *p* < 0.001), and maximum frequency of smoking (*r* = 0.79, *p* < 0.001). Additionally, the agreement between endorsement of any HONC item and continued smoking throughout the period of the longitudinal study (OR = 44, 95% CI, 17–114) indicated the HONC’s predictive validity (5).

HONC has been initially developed to be used among adolescent population (5), then, validated among adult population (11). Furthermore, HONC has shown its performance with several forms of nicotine delivery; cigarettes (5, 11, 12), smokeless tobacco (13), waterpipe (14) and has been recently used also for e-cigarettes use (15–18). Moreover, HONC has proven its efficacy to identify early symptoms of dependence even at low-dose and among occasional smokers (19–21).

In addition to established ND measures such as the Fagerström instruments, the HONC offers a complementary assessment focus. Whereas Fagerström-based scales rely on consumption-related indicators (such as, cigarette quantity, smoking frequency, and time to first cigarette) to index dependence severity (22), the HONC was developed to assess a clearly defined construct-diminished autonomy over tobacco use-using simple, easily interpretable symptom counts and a natural cutpoint (0 symptoms). In a direct comparison with the Fagerström Test for Nicotine Dependence (FTND) in adult smokers, the HONC showed advantages including greater sensitivity to the onset and low levels of dependence and clearer interpretability, and was described as particularly useful when cigarette consumption is low (12). Consistently, evidence suggests that diminished autonomy measured by the HONC can appear very early in the smoking trajectory, including among very infrequent smokers and even after minimal lifetime exposure, often before the onset of daily smoking (23). For this reason, the HONC may be especially relevant when the objective is not only to quantify established dependence severity, but also to detect early loss of autonomy that may be insufficiently captured by consumption-weighted instruments.

Given the emphasized need for valid and reliable instruments adapted to the Moroccan context, and considering the advantageous HONC’s flexibility (applicability to many tobacco forms and different populations), brevity and ease of administration, we consider that it will be practical and valuable for use by clinicians and research investigators. Accordingly, the aim of the present study was to translate and culturally adapt the HONC into Moroccan Arabic and to examine its psychometric properties in a sample of Moroccan smokers. More specifically, we sought to examine its internal consistency, test the fit of its hypothesized one-factor structure, assess item-level psychometric performance using item response theory, and evaluate criterion validity through its association with the Fagerström Test for Cigarette Dependence.

## Methods

### Cross-cultural adaptation

Permission to translate, adapt, and use the HONC was obtained from the scale developer. Subsequently, we carried out the cross-cultural translation and adaptation by referring to the recommendations of Hambleton et al. (24) and Beaton et al. (25).

Two native Moroccan speakers with high proficiency in English (English teachers with over 20 years of experience) were summoned for the translation of the HONC. They independently translated the original HONC into Moroccan dialect. Then, the two versions (T1 and T2) resulting from translations were reviewed in a committee meeting, comprising researchers involved in the study, translators, a psychologist, a psychiatrist, and an epidemiologist. Differences were identified and reduced through discussions in order to produce a consensus version. After that, two other independent translators (having the same profile as the first ones), unaware of the original instrument, did the back translation of the consensus version, so that we obtained two back-translated English versions (BT1 and BT2). A second committee meeting was held to compare BT1 and BT2 with the original version, assess equivalences and whether the items preserved their original meaning. Revisions were made through discussions, and consensus was reached producing an approved pre-final Moroccan version. The latter was, subsequently, subjected to pilot testing among a 20-participant group. And since all items were judged understandable, and nothing was mentioned as ambiguous or confusing, there were no adjustments made to the scale after the pilot test.

### Validation

#### Participants

Sample size adequacy was assessed according to methodological recommendations. A commonly cited rule of thumb for factor analysis recommends a person-to-item ratio of at least 10:1 (26). In addition, other authors have suggested that an overall sample size of 200 to 300 participants is appropriate for factor analysis in general (27). For confirmatory factor analysis with binary data estimated using the WLSMV estimator, previous studies have recommended sample sizes exceeding 200 participants (28, 29), while others have suggested a broader range of 200 to 500 participants (30).

For item response theory, sample size requirements are context -dependent and influenced by multiple factors, including item type, the assumed-response model, dimensionality of the model, etc. (31). Published guidance indicates that, for simple Rasch models, sample sizes as small as 100 may be adequate; however, for more complex IRT models, sample sizes generally range from 200 to 500 (32). In addition, some IRT textbooks provide general recommendations for different models, often suggesting minimum sample sizes of approximatively 250 to 500 respondents (31). Sample size requirements also vary according to the underlying purpose of the analysis: larger samples are needed when extremely accurate item-parameters estimates are desired, whereas smaller samples could be sufficient in preliminary evaluations of questionnaire properties (32).

Based on these guidelines, the sample size in the present study (*N* = 234) was considered adequate for CFA and fell within the lower range reported for IRT analyses.

The validation study was conducted in the addiction treatment center in Fez city. Consecutive current smokers who presented themselves at the center for smoking cessation treatment were recruited. The volunteer smokers were included if they met the following eligibility criteria: (1) current smoking; (2) aged 18 and above; (3) signed the written informed consent. Patients with serious mental illness (such as schizophrenia and bipolar disorder) were excluded, as were participants reporting current use of other psychoactive substances or polysubstance use.

The study period extended from September 2021 to July 2022.

#### Measures

A self-administered anonymous questionnaire was used to collect data. It required between 5 to 10 min for completion. For the limited number of illiterate participants, the questionnaire was filled out by an investigator through a face-to-face interview. The questionnaire consisted of two sections. In the first one, participants were asked to report their sex, age, area of residence, matrimonial status, and educational level, while in the second one, the following scales were implemented:

HONC: which was subject to validation. In the present study, we used the 10-item dichotomously scored version, as was originally conceived. We preferred it to the multiple-choice version, because it is easily applicable and provides direct information as the total score reflects the number of occurring symptoms. Also, in contrast with what is commonly believed, the multiple-choice version of the HONC did not improve the psychometric properties of the scale (10).

FTCD: to investigate criterion validity, we used the Fagerström Test for Cigarette Dependence (FTCD), originally called the Fagerström Test for Nicotine Dependence (FTND) (33, 34), which is intended to provide a measure of nicotine dependence associated with cigarette smoking. The instrument comprises 6 items that assess the number of cigarettes smoked daily (item 1), time to the first cigarette after waking up in the morning (item 2), the cigarette that the smoker would prefer not to give up (item 3), the time of the day when the smoker smokes the most (item 4), the smoker’s inability to abstain from smoking in places where it is prohibited (item 6) and when he is sick (item 5). The three dichotomous items are scored 0 (No) and 1 (Yes), while the other multiple-choice items are scored from 0 to 3. The test’s total score ranges from 0 to 10; with 0 to 2 indicating very low dependence; 3 to 4 indicating low dependence; 5 indicating medium dependence; 6 to 7 indicating high dependence; and 8 to 10 a very high dependence.

We used the test in its Arabic version, translated and validated by Kassim et al. (35).

#### Ethics

The study was approved by the hospital-university ethics committee of Sidi Mohamed Ben Abdellah University (N° 17/21) on April 5, 2022. All participants were informed about the purpose and procedures of the study, their right to decline to participate or withdraw at any time without experiencing any consequences for their access to, timing of, or quality of smoking cessation treatment, and they provided written informed consent. Anonymity and confidentiality of all participants were ensured throughout the study.

### Statistical analysis

The psychometric assessment involved Confirmatory Factor Analysis (CFA) and Item Response Theory (IRT) analysis. All the analyses were performed using R (version 4.5.1; R Core Team, 2025).

First, we provided descriptive statistics for the HONC, in terms of item endorsement rates (means), standard deviations (SD), and corrected item-total correlations, to obtain some preliminary information on participants’ responses and examine how each item correlates to other items in the instrument.

Following this, we analyzed the factor structure of the adapted version of the HONC. Because the HONC has a well-established unidimensional factor structure in its original version and throughout many validation studies (5, 12, 36), we did not perform an exploratory factor analysis. Instead, we conducted a CFA to assess the fit of a single-factor model to the data in our context. This approach is consistent with methodological guidance, as CFA is appropriately used when the factor structure is specified *a priori* based on theory and/ or prior empirical evidence (37, 38). CFA, implemented in the *lavaan* package (v0.6–20) (39), was set up with the Weighted Least Squares Mean- and Variance-Adjusted (WLSMV) estimator for categorical indicators (based on tetrachoric correlations for binary items). The goodness of model fit was evaluated using the relative chi-square (*χ*^2^/df) (values around 3 or lower indicate better fit), CFI (comparative fit index≥ 0.95), TLI (Tucker-Lewis Index ≥ 0.95), RMSEA (root mean square error of approximation ≤0.08) and SRMR (Standardized Root Mean Square Residual ≤0.08) (40, 41). In addition, convergent validity and construct reliability of the latent factor were assessed using average variance extracted (AVE) and composite reliability (CR), calculated from the standardized factor loadings and corresponding error variance values. AVE values ≥ 0.50 were considered indicative of adequate convergent validity, and CR values ≥ 0.70 were considered indicative of satisfactory construct reliability (41).

Criterion validity was tested by assessing the correlation between the HONC’s adapted version total score and the FTCD total score. The association was evaluated using the Spearman’s rank correlation coefficient (Rho). Statistical significance was set at *p* < 0.05. For interpretation, the following criteria were used; correlations were considered negligible (0.00–0.10), weak (0.10–0.39), moderate (0.40–0.69), strong (0.70–0.89), or very strong (0.90–1.00) (42).

As a subsequent step, we evaluated the main IRT assumptions of unidimensionality, monotonicity and local independence.

*Unidimensionality* refers to the fact that only one ability is measured by a set of items that make up the test. However, test performance is always influenced by several cognitive, psychological, and test-taking factors, at least to some extent, making it impossible to strictly meet this assumption. For IRT applications, to sufficiently satisfy the unidimensionality assumption, the presence of a “dominant” factor or component that influences test performance is required (43). In this study, unidimensionality was supported by CFA.

*Monotonicity* means the increasing probability of a higher item score as the level of the latent trait increases, ensuring that item responses meaningfully reflect variations in the underlying construct (44). It was examined using Mokken analysis, with the *mokken* package (v3.1.2) (45). Scalability coefficients (Mokkens’s H) were calculated at the scale (H) and item (Hi) levels. A Loevinger H coefficient < 0.30 was deemed inadequate (46).

*Local independence* is the statistical independence of examinees’ responses to any pair of items when the abilities influencing test performance are held constant (43). Investigation of local independence was done with Yen’s Q3 statistic (47). Adjusted Q3 values greater than 0.20 are considered violations of local independence, according to standard guidelines (48).

After unidimensionality was confirmed, IRT analyses were conducted using the *mirt* package (v1.45.1) (49) in R. To estimate item discrimination (a) and item difficulty (b) parameters for each HONC item, a two-parameter logistic (2PL) model was used. The fit of the 2PL model was compared to the Rasch (1PL) model using the likelihood-ratio test (LR test). Item Characteristic Curves (ICCs), item information functions, and the Test Information Function (TIF) were analyzed to evaluate measurement precision across latent trait levels.

The reliability of the data was assessed through Cronbach’s alpha (*α*) (50) and McDonald’s omega total (ωt) (51), computed from a one-factor model using the *psych* package (v2.5.6) (52). Additionally, IRT-derived marginal reliability was also computed.

## Results

### Background characteristics of participants

The participants’ background characteristics are summarized in Table 1. The mean age was 28.09 ± 9.24 years (range 18–61). Young people (aged 30 or below) constituted more than half of the study population (66.7%). Overall, our sample was predominantly male (more than 85%), single (75%), educated (only 2% who were illiterate) and living in urban area (more than 80%).

**Table 1:** Background characteristics of the participants.

Variable	*n* (%)
Sex	
Male	203 (86.8)
Female	31 (13.2)
Matrimonial status	
Married	41 (17.5)
Single	176 (75.2)
Divorced	17 (7.3)
Widowed	0 (0.0)
Educational level	
Illiterate	5 (2.1)
Primary school	36 (15.4)
Middle/high school	138 (59.0)
University	55 (23.5)
Living area	
Rural	16 (6.8)
Urban	190 (81.2)
Sub-urban	28 (12.0)
	Mean ±SD (range)
Age (in years)	28.09 ± 9.24 (18–61)

### Descriptive statistics

All participants completed the questionnaire. Thus, no missing data were reported.

Before conducting the confirmatory factor analysis, an item-level analysis was performed. Item-level descriptives and point-biserial item–total correlations for all 10 items of the HONC are summarized in Table 2. Endorsement rates (means) ranged from 0.50 to 0.75 (SDs 0.44–0.50).

**Table 2:** Means, SDs and item-total correlations for the HONC items.

Item	Means	SD	Corrected point-biserial item-total *r*
HONC1	0.71	0.46	0.616
HONC2	0.71	0.46	0.640
HONC3	0.71	0.45	0.645
HONC4	0.71	0.46	0.744
HONC5	0.66	0.47	0.713
HONC6	0.50	0.50	0.450
HONC7	0.71	0.46	0.647
HONC8	0.67	0.47	0.687
HONC9	0.71	0.46	0.730
HONC10	0.75	0.44	0.770

Mean = Endorsement rate; SD = standard deviation; Corrected point-biserial item-total *r* = Corrected point-biserial item-total correlation.

Point-biserial item-total correlations were moderate to high, ranging from 0.45 to 0.77 and supported the strength of each items’ association with a common construct. HONC 6 exhibited minimal correlation with the total score (*r* = 0.45), but still within acceptable bounds (values ≥ 0.30 are rated as acceptable) (53).

### Confirmatory factor analysis

**Figure 1:**
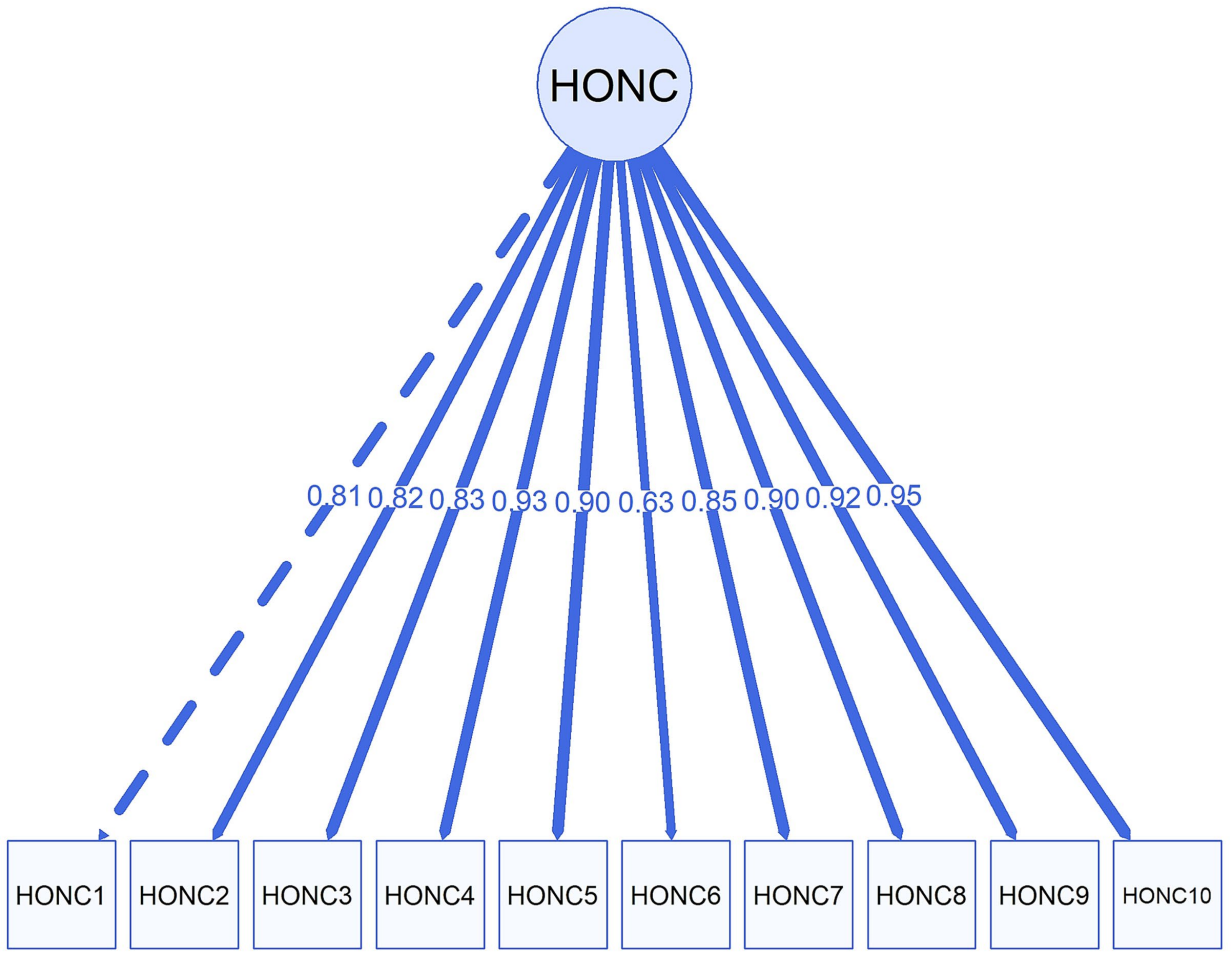
Path diagram of the one-factor CFA model (standardized loadings).

CFA was performed to confirm the unidimensional structure, derived from the conceptual framework underlying the instrument. Estimated using WLSMV estimator on the tetrachoric correlation matrix of the HONC dichotomous items, this analysis showed excellent fit of the model to the data: (*χ*^2^ (35) = 46.71, *p* = 0.089; CFI = 0.998; TLI = 0.998; RMSEA = 0.038 [90% CI 0.000–0.064]; SRMR = 0.059). The CFA model’s path diagram is provided in Figure 1.

As reflected in Table 3, standardized factor loadings were uniformly strong, ranging from 0.629 (HONC 6) to 0.952 (HONC 10) and all estimated loadings were statistically significant (*p* < 0.001), indicating that all items converge on the latent construct at adequate levels. A rule of thumb is that standardized loading estimates should be 0.5 or higher (41).

**Table 3:** CFA results for the one-factor HONC model.

Item	B (Unstd.)	SE	*z*	*p*	Std. loading	*R* ^2^
HONC1	1.000	–	–	–	0.809	0.655
HONC2	1.009	0.065	15.59	<0.001	0.817	0.667
HONC3	1.029	0.061	16.83	<0.001	0.833	0.693
HONC4	1.146	0.060	19.03	<0.001	0.927	0.860
HONC5	1.106	0.061	18.15	<0.001	0.895	0.801
HONC6	0.777	0.074	10.57	<0.001	0.629	0.396
HONC7	1.045	0.069	15.15	<0.001	0.845	0.715
HONC8	1.118	0.064	17.49	<0.001	0.905	0.819
HONC9	1.136	0.065	17.47	<0.001	0.919	0.845
HONC10	1.176	0.068	17.34	<0.001	0.952	0.906

B = unstandardized loading; SE = standard error; *z* = Wald z statistic; *p* = two-tailed *p*-value; Std. Loading = standardized factor loading; *R*^2^ = squared multiple correlation. Estimator: WLSMV (robust DWLS). All estimated loadings were significant (*p* < 0.001).

Convergent validity and composite reliability of the one-factor model were further supported by an AVE of 0.736 and a CR of 0.965, exceeding the recommended thresholds of 0.50 and 0.70, respectively.

### Criterion validity

Significant moderate positive correlation was found between HONC total score and FTCD total scores (Spearman’s *ρ* = 0.57, *p* < 0.001), showing the concordance between the two measures (higher levels of loss of autonomy over tobacco use as measured by the HONC, were associated with higher levels of nicotine dependence assessed by the FTCD), and supporting criterion validity.

### Item response theory

#### Assumptions’ check

Prior to conducting IRT analyses, we tested its three assumptions; (a) unidimensionality, (b) local independence, and (c) Monotonicity.

##### Unidimensionality

The one-factor CFA model estimated with WLSMV fit the tetrachoric correlations well (*χ*^2^ (35) = 46.71, *p* = 0.089; CFI = 0.998; TLI = 0.998; RMSEA = 0.038 [90% CI 0.000–0.064]; SRMR = 0.059), and all standardized loadings were ≥ 0.60, indicating that the items are adequately represented by a single latent dimension; thus, the unidimensionality assumption is fulfilled.

##### Monotonicity

Table 4 results show strong Mokken scalability for the overall scale (H = 0.557, SE = 0.037), which indicated the scale is strong. According to Mokken, a scale is considered weak if 0.30 ≤ H < 0.40, a medium scale if 0.40 ≤ H < 0.50, and strong if H ≥ 0.50. Moreover, Hi > 0.30 (acceptability threshold) was achieved for all the items of the HONC (Hi = 0.496–0.663) (46). Therefore, the scale can be safely treated as unidimensional and can order the respondents on the latent continuum. Besides, as illustrated by the #vi column, no monotonicity violations were detected for any HONC item, meaning that the probability to endorse an item increases as the latent trait “loss of autonomy” increases. This meets the monotonicity requirement for unidimensional IRT.

**Table 4:** Mokken scalability and monotonicity diagnostics for HONC items.

Item	Hi	SE	#vi	#zsig
HONC1	0.496	0.047	0	0
HONC2	0.515	0.049	0	0
HONC3	0.522	0.054	0	0
HONC4	0.589	0.039	0	0
HONC5	0.595	0.041	0	0
HONC6	0.522	0.058	0	0
HONC7	0.520	0.048	0	0
HONC8	0.571	0.042	0	0
HONC9	0.579	0.040	0	0
HONC10	0.663	0.053	0	0
Scale H	0.557	0.037		

Hi = item scalability (Loevinger’s Hᵢ); #vi = number of monotonicity violations; #zsig = number of statistically significant violations.

##### Local independence

We examined local independence using Yen’s Q3 statistic. It is the linear correlation between the residuals of each pair of items. There should be essentially no correlation between the residuals of two distinct items if participant ability is the only latent feature that determines the likelihood of correctly answering items. Item pairs with a value of Q3 > 0.20 should be flagged, according to Yen and Fitzpatrick. A high residual correlation might indicate that the response to one item influences the response to the other, or both items measure another unintended construct (54, 55).

The highest positive Q3 found was 0.196 for item pair HONC 8-HONC 9 (Table 5), which is below the standard 0.20 threshold. No item pairs exceeded the Yen’s Q3 cutoff, supporting the local independence assumption.

**Table 5:** Highest residual correlations (Yen’s Q3).

Pair	Q3
HONC8-HONC9	0.196
HONC7-HONC8	0.124
HONC7-HONC10	0.119
HONC1-HONC3	0.118
HONC1-HONC2	0.115

#### Item response theory analysis

##### Model specification and rationale

For dichotomous items, IRT typically offers three common models: the one-parameter logistic model (1PL, Rasch), the two-parameter logistic model (2PL), and the three-parameter logistic model (3PL). They distinguish themselves based on the number of estimated parameters. A higher model in nested IRT models has at least one extra parameter in addition to all of the lower model’s parameters. The likelihood-ratio test (LR test) may be used to assess the relative fit of nested unidimensional models, and the best suited model is employed (56, 57).

Though conceptually more comprehensive, the 3PL model adds another parameter “the probability for guessing” that is limited to multiple-choice cognitive tests where test takers can guess the right answer. The 3PL model is not conceptually adequate and was not further investigated because the HONC consists of self-report behavioral symptoms without guessing effects (55).

One-parameter (Rasch) and two-parameter logistic models were estimated, and their relative fit was compared using a likelihood-ratio (LR) test implemented through ANOVA function applied to the fitted models.

As can be seen in Table 6, the 2PL model outperformed the 1PL model in terms of fit (Δ*χ*^2^(9) = 43.85, *p* < 0.001), suggesting that item discrimination parameters differ significantly between items. Several fit indices showed the improvement (AIC, SABIC, HQ). As a result, the 2PL model was kept for the analyses that followed.

**Table 6:** Comparison of two IRT model fit indices (Rasch and 2PL).

Model	LogLik	AIC	SABIC	HQ	BIC	Loglikelihood ratio test
1PL	−1026.983	2075.965	2079.109	2091.290	2113.974	–
2PL	−1005.056	2050.112	2055.828	2077.976	2119.219	*χ*^2^ (9) = 43.853, *p* < 0.001

AIC, Akaike Information Criterion; SABIC, Sample-size Adjusted Bayesian Information Criterion; HQ, Hannan–Quinn information criterion; BIC, Bayesian Information Criterion (Schwarz); logLik, Log-likelihood of the model; *χ*^2^, Chi-squared statistic; df, degrees of freedom. *Lower AIC, SABIC, BIC and HQ values indicate a better-fitting model (63).

Although the 2PL model’s AIC, SABIC, and HQ values were lower (better) than those of the 1PL model, its BIC value was slightly higher, which is expected given the higher penalty that BIC gives to the number of parameters (BIC tends to choose the simpler model) (57). In our case, because three of the four indices favored the 2PL and the likelihood-ratio *χ*^2^ test indicated a significantly better fit for the 2PL over the 1PL model, the 2PL model was retained.

##### Item parameter estimates

The discrimination (*a*) and difficulty (*b*) parameters of the 2PL IRT model for the 10 items are shown in Table 7. Discrimination reflects the steepness of the item characteristic curve in its middle section; the steeper the curve, the more effectively the item may differentiate across people with varying latent trait levels: flatter curves, on the other hand, suggest weak discrimination. Item discrimination levels are classified as very low at 0.01–0.34; low at 0.34–0.64; moderate at 0.65–1.34; high at 1.35–1.69; and very high at 1.70 and above (58).

**Table 7:** Item response theory parameters estimate of HONC items.

Item	Discrimination (a)	Difficulty (b)
HONC1	2.215	−0.639
HONC2	2.371	−0.635
HONC3	2.517	−0.634
HONC4	4.220	−0.501
HONC5	3.679	−0.392
HONC6	1.401	0.003
HONC7	2.596	−0.610
HONC8	3.249	−0.423
HONC9	3.697	−0.521
HONC10	4.694	−0.625

All HONC items significantly contributed to differentiating participants along the loss of autonomy continuum, as evidenced by the generally high discrimination values in our data, which ranged from 1.40 (HONC6) to 4.69 (HONC10), with particularly strong discrimination for HONC4, HONC5, HONC9, and HONC10 (*a* ≥ 3.50).

The difficulty parameter represents the point on the latent continuum at which the probability of endorsing an item is 0.5 (58). HONC items showed a difficulty parameter ranging from −0.64 to 0.00 (low to moderate difficulty range). All items, except item 6, demonstrated negative *b* values; indicating that they are endorsed even at relatively low levels of loss of autonomy, while item 6 (*b* = 0.00) requires slightly higher level of the trait. Overall, the parameter estimates indicate that HONC items are effective at capturing early stages of loss of autonomy, which fits the original purpose of the scale, while still offering strong discrimination between individuals with different trait levels.

##### Item characteristic curves

**Figure 2:**
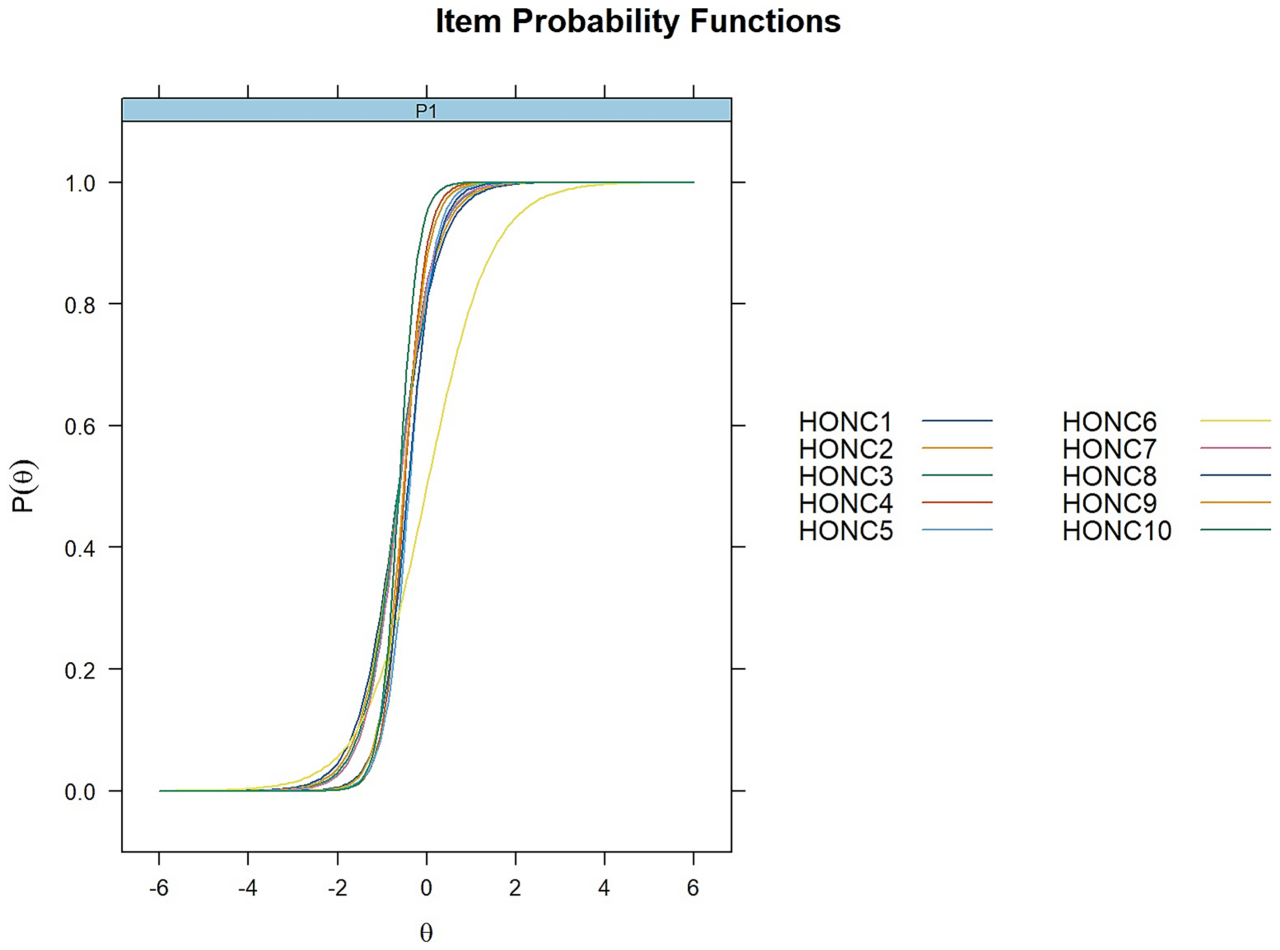
Item characteristic curves (ICCs) for the HONC items (2PL model).

**Figure 3:**
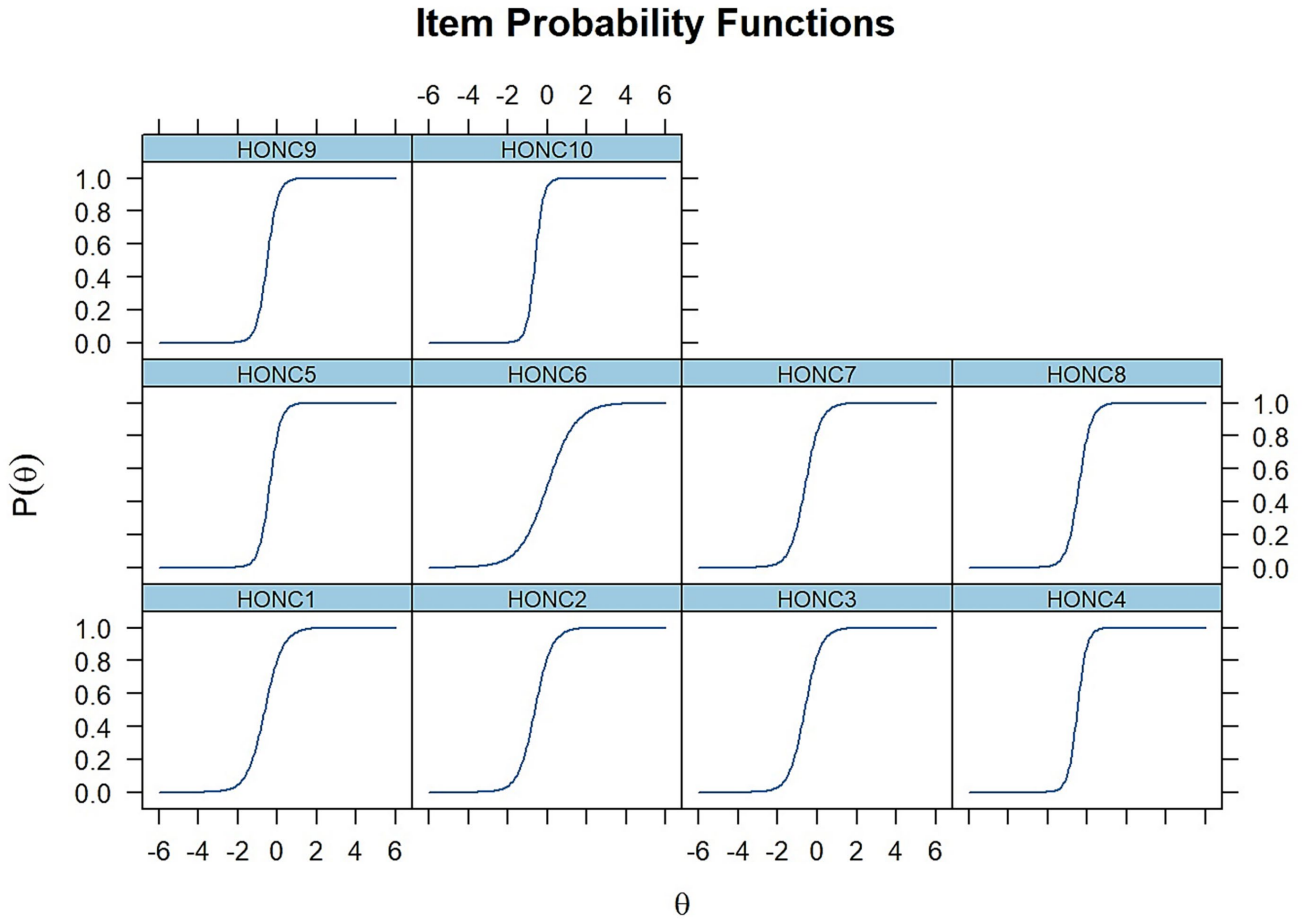
Item-level item characteristic curves for the HONC items (2PL model).

Item Characteristic Curves were plotted for the 10 HONC items, to visually present the relationship between the latent level of loss of autonomy (*θ*) (X-axis) and probability endorsing each HONC item (Y-axis), under the 2-PL model. As seen in Figures 2, 3, all ICCs have the expected sigmoidal shape, indicating that probability of endorsing each item monotonically increases as the latent trait of the respondent becomes higher. Items with higher *a* values (e.g., HONC10, HONC4, HONC9, and HONC5) show steeper curves around their difficulty locations, reflecting higher capacity to detect subtle differences in the loss of autonomy level of respondents. On the contrary, HONC6 has a comparatively flatter curve which is consistent with its lower discrimination estimate, even if it still exhibits appropriate monotonic functioning and an informative contribution to the scale.

**Figure 4:**
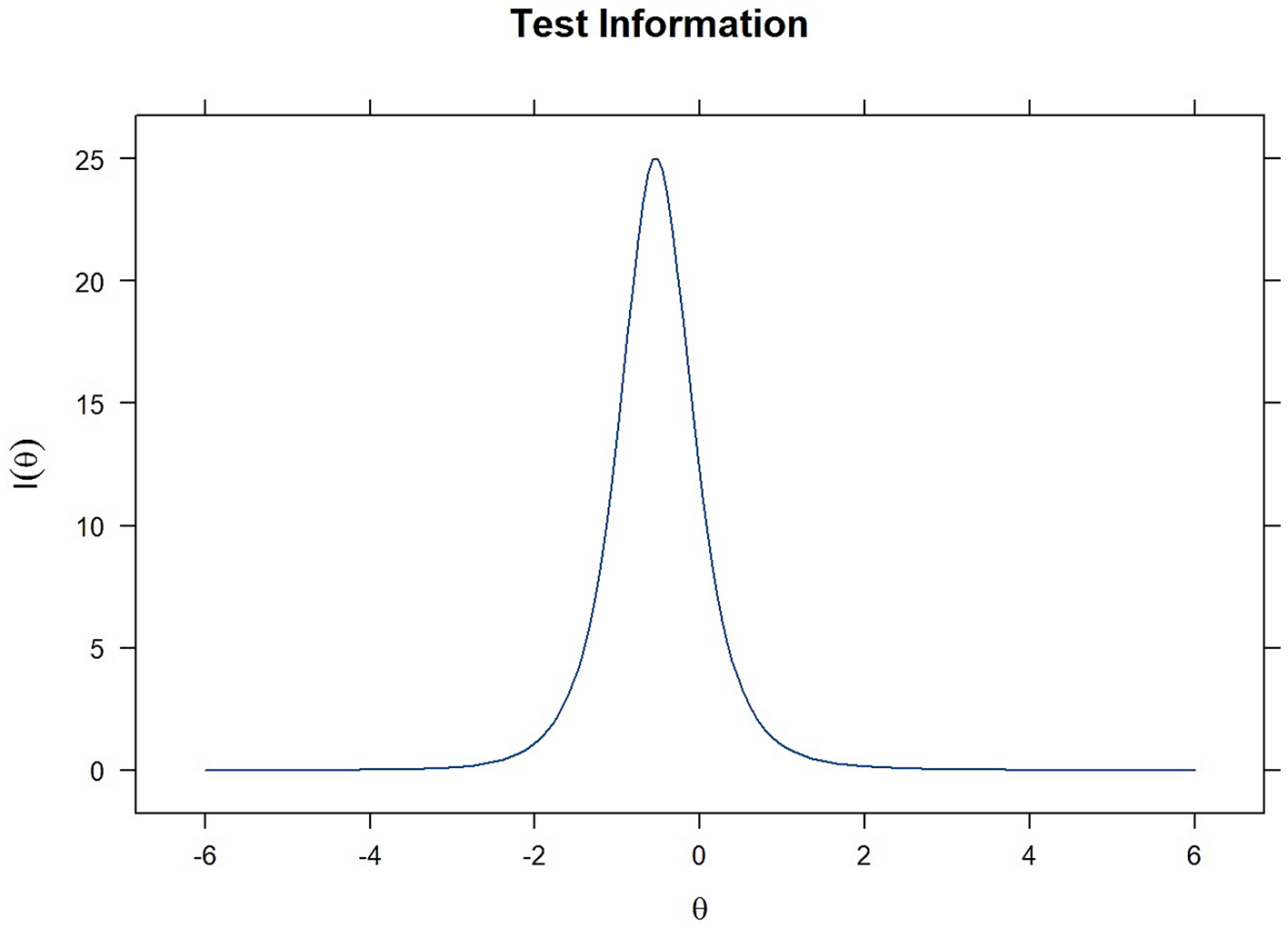
Test information function of the HONC (2PL model).

While ICCs demonstrate the functionality of individual items throughout the latent continuum, the Test Information Function (TIF) offers a direct evaluation of the measurement accuracy of the HONC at various levels of loss of autonomy. According to the TIF (Figure 4), the HONC provides maximum measurement precision at *θ* = −0.52, where the information function peaks at 25.05. This indicates that the scale is most precise for respondents with low to moderately low levels of the latent trait (individuals presenting early or moderate manifestations of loss of autonomy over tobacco use). Precision decreases toward both extremes of θ, indicating lower measurement accuracy outside this range. Overall, the shape of the curve is consistent with the HONC’s intended use for detecting early manifestations of ND.

### Reliability

The reliability analysis of the HONC revealed good internal consistency: Cronbach’s alpha = 0.905, McDonald’s omega = 0.908. Additionally, the total scale demonstrated IRT marginal reliability of 0.75.

## Discussion

This study evaluated the psychometric properties of the Moroccan Arabic version of HONC, which was designed to assess “loss of autonomy over tobacco use,” using CFA and IRT. The findings converged to indicate that the scale is a reliable and psychometrically sound tool.

The CFA supported the hypothesized one-dimensional structure of the Moroccan Arabic version of the HONC. All items loaded significantly (>0.60) on the latent factor “loss of autonomy over tobacco use.” The one-factor model showed excellent fit to the data (*χ*^2^ (35) = 46.71, *p* = 0.089; CFI = 0.998; TLI = 0.998; RMSEA = 0.038 [90% CI 0.000–0.064]; SRMR = 0.059). This aligns with the factor structure of the original version, revealing one principal factor accounting for 66% of the total variance (5). Furthermore, other studies conducted among teenagers and young adults in different cultural contexts have corroborated the one-factor structure. A previous study conducted among adult smokers (11) have shown that the checklist is essentially unidimensional, reflecting a single latent construct of loss of autonomy over tobacco use. A similar conclusion was reached in a study aiming to explore psychometric properties of the HONC, involving college students who were current smokers, where the HONC was found to measure a single underlying dimension (59). Evidence from a factor analysis of the HONC in a sizable sample of adult smokers also supports this model (12). More recently, a validation study of the HONC among Spanish adolescents (36) has also replicated this factor structure.

The criterion validity was supported by the statistically significant positive correlation between Moroccan HONC and FTCD total scores (*ρ* = 0.57, *p* < 0.001), indicating that the adapted version of HONC aligns with an established measure of nicotine dependence (gold standard).

Using IRT analyses, we were able to thoroughly understand the item-level function. Each item was analyzed for its difficulty and discrimination values. The discrimination parameter (*a*) values varied across items, indicating the scale’s heterogeneous item functioning. Several items demonstrated very high discrimination estimates (e.g., HONC10, *a* = 4.69; and other items with *a* > 3.5). While these values may reflect a strong capacity to distinguish between respondents with close levels of nicotine dependence in this sample, discrimination parameters are known to be sensitive to sampling variability in small-to-moderate samples (60, 61); therefore, very high *a* estimates may be partially inflated at *N* = 234. Accordingly, the magnitude of the largest discrimination parameters should be interpreted cautiously, and replication in larger samples is warranted. Importantly, our conclusions do not rely solely on items’ discrimination estimates; they are supported by (1) test-level precision showing where the HONC measures most accurately (TIF/SE(*θ*)) and (2) external convergent evidence, namely the observed association between the HONC total scores and FTCD scores. HONC6 showed the lowest discrimination estimate (*a* = 1.40), which is congruent with its comparatively weaker item–total correlation and its lower factor loading in CFA. This pattern is logically interpretable. HONC6 (“Is it hard to keep from smoking in places where you are not supposed to, like school?”) is conceptually different from the other HONC items, because it represents “situational constraints,” that are external environmental regulations (in this case; school rules, enforcement, supervision, etc.). Therefore, endorsement of this context-dependent item is dependent not only on the internal loss of autonomy, but also on the “external” contextual opportunities to violate smoking restriction, which can differ between environments and cultures. Because of this, HONC6 showed lower endorsement rates (0.50 in our study) compared to the other items, reflecting more “internal” loss of autonomy; some previous studies have noted this pattern, as well (12, 36). Importantly, despite having a reduced loading, HONC6 maintained a positive correlation with the latent factor and did not violate the assumptions of monotonicity or local independence, which supports its retention as a relevant but context-sensitive indicator of nicotine dependence.

The difficulty parameters *(b)* were primarily situated in the negative to near-zero spectrum, signifying that—within this sample—the majority of HONC items are endorsed at lower to moderate levels of the latent trait. However, interpretation of item “easiness” must consider the sampling frame. Indeed, although IRT parameters are theoretically sample-invariant under correct model specification, in practice, parameter estimates and their interpretation may be influenced by the population studied and its trait distribution (62). In this context, because participants were treatment-seeking smokers recruited from an addiction treatment center, the sample likely reflects a higher dependence distribution than would be expected in general populations, which may increase symptom endorsement and shift estimated difficulty values downward. Therefore, the present calibration should not be taken as definitive evidence that the items are universally “easy” or that the scale is optimally targeted to the earliest stages of dependence across populations. Instead, the results should be interpreted as reflecting both the HONC’s intended focus on early loss of autonomy and the higher-severity distribution of the treatment-seeking sample. Consequently, replication in community-based and lighter-smoking samples is needed to determine whether these *b* estimates generalize.

Regarding reliability, the scale demonstrated strong internal consistency, with Cronbach’s *α* = 0.905 and McDonald ωt = 0.908, exceeding widely accepted thresholds (27). Furthermore, the marginal reliability coefficient obtained under IRT yielded a value of 0.75, which is an acceptable average precision for a brief screening tool. The test information function (TIF) reached a maximum value of approximatively 25.05 at *θ* ≈ −0.52, showing high measurement precision at low to moderately low trait levels. In line with this pattern, the standard error of measurement reached its lowest value around θ ≈ −0.52, and increased progressively toward more extreme trait levels. Considered together, these findings support that the Moroccan Arabic HONC is highly reliable overall, with optimal precision at lower to moderate levels of nicotine dependence, showing that it is optimally informative for detecting emerging loss of autonomy over tobacco use, rather than for discriminating among users with extremely high levels of nicotine dependence.

The findings of this study have to be seen in the light of several limitations. First, the study’s cross-sectional nature poses limitations regarding conclusions about the temporal stability of the adapted version of the HONC. Second, the exclusive reliance on a sample of smokers from an addiction treatment center may have limited the generalizability of the findings to other population samples. Third, the sample was disproportionately male (about 87%), which could have introduced sex bias This imbalance reflects established gender disparities in smoking prevalence and treatment seeking behavior in the study setting, but may constrain the findings’ generalizability to females. Fourth, all data were collected via self-reported questionnaires, which are prone to recall and social desirability biases. Fifth, although the sample size (*N* = 234) was acceptable for the categorical CFA, it lies at the lower end of commonly recommended ranges for 2PL IRT estimation; therefore, item parameter estimates -particularly very high discrimination values—should be interpreted with appropriate caution and confirmed in larger samples.

## Conclusion

In conclusion, this study provides researchers with a Moroccan Arabic-translated and culturally adapted version of the HONC. It demonstrated good psychometric properties; it is, thus, a valid and reliable instrument to screen nicotine dependence. The HONC appears to be well suited for screening purposes, particularly in identifying individuals with low to moderate nicotine dependence. Added to its shortness and ease to administer, this reinforces its suitability for early detection programs, school-based surveys and public health monitoring.

## Data Availability

The raw data supporting the conclusions of this article will be made available by the authors, without undue reservation.
